# HEALTH CONDITIONS AND CONTINUOUS POSITIVE AIRWAY PRESSURE DISCONTINUATION IN US MIDDLE-AGED AND OLDER ADULTS

**DOI:** 10.1093/geroni/igae098.0254

**Published:** 2024-12-31

**Authors:** Vishaldeep Sekhon, David Roth, Jennifer Albrecht, Emerson Wickwire, Adam Spira, Halima Amjad, Chien-Yu Tseng, Christopher Kaufmann

**Affiliations:** Johns Hopkins University, Baltimore, Maryland, United States; Johns Hopkins University, Baltimore, Maryland, United States; University of Maryland School of Medicine, Baltimore, Maryland, United States; University of Maryland School of Medicine, Baltimore, Maryland, United States; Johns Hopkins University, Baltimore, Maryland, United States; Johns Hopkins University School of Medicine, Baltimore, Maryland, United States; University of Florida College of Pharmacy, Gainesville, Florida, United States; University of Florida College of Medicine, Gainesville, Florida, United States

## Abstract

Although continuous positive airway pressure (CPAP) is an efficacious treatment for obstructive sleep apnea (OSA), suboptimal adherence is common, reducing its effectiveness. OSA co-occurs with chronic health conditions that may impact adherence to CPAP. This study evaluated health conditions as predictors of CPAP discontinuation over 2 years. Data came from the 2016 and 2018 Health and Retirement Study, a longitudinal cohort study of a nationally representative sample of 20,000 older adults in the U.S. In both years, participants were asked if “a doctor had ever told them they have a sleep disorder,” and if so, which disorder and any treatments received (including CPAP). In 2016, participants also reported whether a doctor ever told them they had several health conditions (high blood pressure, diabetes, cancer, lung disease, heart disease, stroke, psychiatric problems, and arthritis). We identified CPAP users in 2016 with available data in 2018. Participants not listing CPAP in 2018 were considered to have discontinued CPAP. We used logistic regression to determine which health conditions predicted CPAP discontinuation after controlling for age, sex, race/ethnicity, education, BMI, and marital status. Of N=902 CPAP users in 2016, 38% did not indicate CPAP use in 2018. Participants diagnosed with diabetes in 2016 had 36% increased odds of CPAP discontinuation (AOR=1.36, 95% CI=1.02-1.80) compared to those without diabetes after accounting for other covariates. CPAP discontinuation did not differ across other health conditions. Overall, CPAP users with diabetes were at increased likelihood of CPAP discontinuation, necessitating efforts to encourage CPAP adherence in this vulnerable group.

